# Climatic conditions and child height: Sex-specific vulnerability and the protective effects of sanitation and food markets in Nepal

**DOI:** 10.1016/j.ehb.2016.07.002

**Published:** 2016-12

**Authors:** Prajula Mulmi, Steven A. Block, Gerald E. Shively, William A. Masters

**Affiliations:** a Friedman School of Nutrition Science and Policy, Tufts University, 150 Harrison Avenue, Boston, MA 02111, United States; b Fletcher School of Law and Diplomacy, Tufts University, 160 Packard Avenue, Medford, MA 02155, United States; c Department of Agricultural Economics, Purdue University, 403 West State Street, West Lafayette, IN 47907, United States; d Department of Economics, Tufts University, 8 Upper Campus Road, Medford, MA 02155, United States

**Keywords:** Seasonality, Child health, Child nutrition, Maternal health, Sanitation

## Abstract

Environmental conditions in early life are known to have impacts on later health outcomes, but causal mechanisms and potential remedies have been difficult to discern. This paper uses the Nepal Demographic and Health Surveys of 2006 and 2011, combined with earlier NASA satellite observations of variation in the Normalized Difference Vegetation Index (NDVI) at each child’s location and time of birth to identify the trimesters of gestation and periods of infancy when climate variation is linked to attained height later in life. We find significant differences by sex: males are most affected by conditions in their second trimester of gestation, and females in the first three months after birth. Each 100-point difference in NDVI at those times is associated with a difference in height-for-age *z*-score (HAZ) measured at age 12–59 months of 0.088 for boys and 0.054 for girls, an effect size similar to that of moving within the distribution of household wealth by close to one quintile for boys and one decile for girls. The entire seasonal change in NDVI from peak to trough is approximately 200–300 points during the 2000–2011 study period, implying a seasonal effect on HAZ similar to one to three quintiles of household wealth. This effect is observed only in households without toilets; in households with toilets, there is no seasonal fluctuation, implying protection against climatic conditions that facilitate disease transmission. We also use data from the Nepal Living Standards Surveys on district-level agricultural production and marketing, and find a climate effect on child growth only in districts where households’ food consumption derives primarily from their own production. Robustness tests find no evidence of selection effects, and placebo regression results reveal no significant artefactual correlations. The timing and sex-specificity of climatic effects are consistent with previous studies, while the protective effects of household sanitation and food markets are novel indications of mechanisms by which households can gain resilience against adverse climatic conditions.

## Highlights

• In Nepal, the effects of climatic conditions on child height depend on the timing of exposure.
• For boys, child height is linked to climate during pregnancy, and for girls, in early infancy.
• Household sanitation (toilets) and food markets eliminate correlations between climatic conditions and child height.
• Better household sanitation could protect children from the effects of climate by blocking disease transmission.
• Food markets could protect against local climatic variation by regulating dietary intake.

## 1 Introduction and motivation

Attained height is among the most important indicators of childhood health or deprivation. Approximately 25 percent of each year’s worldwide cohort of infants grow up to be stunted, and the dietary or disease conditions that limit linear growth in childhood also contribute to poor educational attainment, low earnings, and high mortality rates later in life (IFPRI, 2014, UNICEF, 2015).

Stunting rates have been especially high in Nepal, where extreme poverty and political instability led to rates as high as 57 percent in 2001, before declining to 41 percent in 2011 (United Nations, 2013). Despite this improvement, Nepal remains one of the 10 countries in the world with the highest stunting prevalence (UNICEF, 2014), making it a high-priority location for research into increasingly effective ways of protecting children from harmful early-life circumstances.

Socioeconomic factors associated with stunting in Nepal are described by Headey and Hoddinott (2015), who show how changes in household and community-level characteristics help explain local variation and the overall improvement in this indicator from 2001 to 2011. Key changes involved both greater household sanitation and access to improved diets, which are pillars of the Nepal government’s multisector nutrition plan (Nepal NPC, 2012). Despite this progress, however, poor sanitation and inadequate food intake remain widespread and are likely to be worsened by rising temperatures and more variable rainfall associated with climate change (IPCC, 2014).

This paper uses satellite data on vegetation near each child’s home as an indicator of changing agroclimatic conditions, with randomness in the month of birth providing a natural experiment in the timing of exposure to more or less advantageous circumstances. Our use of variation in birth timing relative to changes in climatic conditions contribute to a rapidly growing body of literature using natural experiments to study the determinants of human health (Angrist and Krueger, 2001, Akresh et al., 2011, Lokshin and Radyakin, 2012, Tiwari et al., 2013, Brown et al., 2014), addressing the timing and mechanisms by which early conditions influence later outcomes (Skoufias and Vinha, 2012, Kumar et al., 2016, Schultz-Nielsen et al., 2016).

Our identification strategy takes a difference-in-differences approach, testing whether household sanitation and district-level food markets can protect children against the health consequences of unfavorable agroclimatic conditions at sensitive times in their early growth and development. The specific data we use are the Nepal Demographic Health Survey (NDHS) for child health, sanitation, and other household characteristics from 2006 and 2011, combined with Normalized Difference Vegetation Index (NDVI) data from the National Aeronautics and Space Administration (NASA) for 2000–2012 at each child’s location, and the Nepal Living Standard Survey (NLSS) to characterize local agricultural markets for 2003–2004 and 2010–2011.

By combining three kinds of data, we are able to identify patterns in attained heights of children observed at 12–59 months of age, and test whether sanitation and food markets limited their association with agroclimatic conditions experienced during gestation and the first year after birth. We find the underlying patterns to be sex-specific, with systematic differences in how later heights relate to NDVI fluctuations that occurred during infancy and pregnancy. These differences are consistent with both gender bias in infant care (Maccini and Yang, 2009) and physiological differences in fetal development before the sex of the child is known (DiPietro and Voegtline, 2015, Rosenfeld, 2015). We find that improved household sanitation and more commercialized food markets limit both kinds of vulnerability, providing significant protection from agroclimatic conditions for both pregnant mothers and infants.

## 2 Background and identification strategy

### 2.1 Agriculture and climate in Nepal

Nepal is a landlocked country with a population of approximately 27 million people, of whom about 85 percent live in rural areas (Nepal MoHP, 2012) and are highly reliant on rain-fed agriculture (Nepal MoAD, 2013). The country features three distinct ecological zones: Mountains (52,000 km^2^), Hills (61,000 km^2^), and Terai or lowlands (34,000 km^2^), with varying population densities. The Mountain zone has a dry alpine climate and is situated at the highest altitude (>2500 m), with steep and rugged terrain and short growing seasons. The Hills have a mostly temperate climate (500–2500 m), and the Terai (<500 m) has a mostly subtropical and humid climate (Nepal MoHP 2012). Although the Terai occupies 23 percent of the country’s landmass, it hosts almost half of the population (48 percent) and most of the cultivable land (56 percent) (Nepal MoAD, 2013). The most commonly grown crops are cereals including maize, millet, barley, rice, and wheat (WFP, 2014). Consistent with global trends of increasing temperature and erratic rainfall patterns (NASA, 2015), temperatures in Nepal increased by 1.5 °C over the period from 1978 to 2005 (Krishnamurthy et al., 2013), while rainfall declined in frequency and increased in intensity (Malla, 2008).

The impacts of climate trends and fluctuations can be seen through changes in sowing dates, crop duration, crop yields, and management practices (IPCC, 2014). Between 1978 and 2008, the summer months (May–August) became increasingly hot and wet, and winter months (November–February) became colder and drier. During that time, the higher levels of rainfall in summer increased rice yields but decreased yields for other crops, while lower levels of rainfall in winter decreased maize yields (Joshi et al., 2011). For this paper, we use NDVI data to summarize the complex pattern of variation in both rainfall and temperature, providing a simple index of changing agroecological conditions in the area around each child’s home.

### 2.2 Seasonality and child nutrition

Seasonal variation and other climatic changes have a clear link to the nutritional status of children in many contexts, even in industrialized countries (Chodick et al., 2009). In the UK, for example, babies born in winter have significantly lower birth weights, educational attainment, and adult heights, perhaps as a result of low vitamin D levels during early life (Day et al., 2015). In the United States, children conceived in the summer have a higher prevalence of birth defects (McKinnish et al., 2014) and different genetic characteristics (Rietveld and Webbink, 2016). Some seasonal patterns may be the result of selection effects, as Buckles and Hungerman (2013) show in their study of winter births in the United States which occur disproportionately among disadvantaged youths. However, in developing countries, studies have repeatedly found relatively large agroclimatic patterns that cannot be explained by selection effects. For example, in the Democratic Republic of Congo, Darrouzet-Nardi (2015) shows that children born during wet seasons grow up to be shorter, with no evidence for selection effects of adverse birth timing of children with lower levels of household wealth or education.

Agroclimatic fluctuations may affect child nutrition through both disease risk and dietary intake. A principal source of variation in both kinds of risk is rainfall: children born during monsoon months in India have lower height and weight than children born during the fall–winter months (Lokshin and Radyakin, 2012). In addition, rainfall fluctuations in Indonesia have been shown to affect child health in both rural and urban areas (Yamauchi, 2012, Cornwell and Inder, 2015). These associations often depend on the timing of exposure. For example, Tiwari et al. (2013) show that in Nepal, a child’s weight for age is positively correlated with rainfall in the previous monsoon season, but negatively correlated with rainfall in the current monsoon. Temperature may play an independent role, as suggested by Hu and Li (2016), among others, although Nepal’s complex topography complicates efforts to analyze the effects of spatial variation in temperature. In any case, covariance among climatic variables, agricultural conditions, and dietary intake makes it difficult to distinguish one factor from another. In Malawi, for example, the prevalence of underweight among children under age five rises during the rainy season, which is also the preharvest period, when maize prices are highest (Sassi, 2015).

### 2.3 Vulnerability in utero and after birth

Gestation and the first two years after birth are the most critical periods for child development, and have clear impacts on physical, cognitive, and other outcomes later in life (Almond, 2006, Black et al., 2013, Hoddinott et al., 2013). Adverse conditions in utero and during the first two years of life can cause high perinatal mortality and subsequent stunting (Coffey, 2015) as well as low weight and anemia (Kumar et al., 2016), low economic productivity (Paxson and Schady, 2007, Hoddinott et al., 2013), and less academic success (Schultz-Nielsen et al., 2016). Affected children may also have higher odds of developing cardiovascular disease and other conditions (Popkin et al., 1996, Sawaya et al., 2003), as set forth in the fetal origins hypothesis of Barker (1995).

This paper expands on the previous literature by using quarterly variation in NDVI to identify sex-specific differences in the timing of vulnerability before and after birth, and to test for the possible protective effects of improved sanitation and food markets. Our focus on sex differences follows that of Skoufias and Vinha (2012), and our attention to the exact timing of exposure follows Andalon et al. (2014) and Carlson (2015), among others, building on previous work in South Asia using Demographic and Health Surveys (DHS) in both India and Nepal that suggest a stronger link between rainfall variation and child height during early months in infancy than during other periods of a child’s life (Lokshin and Radyakin, 2012, Tiwari et al., 2013). Moreover, focusing on the growing seasons in Nepal, other researchers find that anomalies in vegetation density show higher correlations with stunting during the in utero and infancy phases than in other phases of a child’s development (Shively et al., 2015).

The timing and magnitude of vulnerability to agroclimatic conditions could differ by the child’s sex. There is a growing body of evidence suggesting differential effects of prenatal stress on male and female fetuses on perinatal outcomes (Aibar et al., 2012, Mulla et al., 2013, Persson and Fadl, 2014) and adult health (Scholte et al., 2015). In general, female fetuses are more resilient and adaptive to stress than are male fetuses (DiPietro and Voegtline, 2015, Rosenfeld, 2015). Evidence from studies that examined the effects of stressors such as intrauterine lead and pesticide exposure, and maternal alcohol and drug use, suggest that exposed male fetuses are more likely to be born preterm and have poorer scores on developmental assessments than females (Rosenfeld, 2015). Historical data from Danish cemeteries suggest that males’ heights increased during the 19th century while females’ did not (Jørkov, 2015). In malnourished populations today, on average boys are more likely to be stunted than girls, but after birth, when the child’s sex is known, gender discrimination may play an important role in health outcomes. For example, Maccini and Yang (2009) show that Indonesian families commonly protected boys more than girls from early-life shocks, and countries with more gender discrimination in favor of boys have lower rates of stunting in males relative to females (World Bank, 2008) with intergenerational effects (Osmani and Sen, 2003).

### 2.4 Protective effects of sanitation and food markets

Numerous interventions could provide protection against the disease transmission and dietary inadequacy associated with agroclimatic conditions. In this paper, we study two kinds of variables that are of particular interest to policymakers: household sanitation and local food markets. Sanitation has become an increasingly important policy tool in recent years, especially for South Asia (Hammer and Spears, 2016), while the impact of agricultural commercialization on nutrition has been of longstanding concern around the world (Von Braun and Kennedy, 1994).

The potential efficacy of household sanitation against stunting operates through preventing fecal–oral transmission of diseases such as diarrhea and enteropathy, which cause both mortality and stunting through loss of nutrients, decreased absorption of nutrients, and a weakened immune system (Checkley et al., 2008; Humphrey 2009). There may also be selection effects, as households with toilets may have other favorable conditions for child development, but the prevention of disease transmission provides a clear causal mechanism to explain how sanitation might improve nutritional status (Coffey and Geruso, 2015) and linear growth (Checkley et al., 2004, Lin et al., 2013). In this study, we focus on the average effects on each child of having a toilet in their own household; future work could address the externalities among households described by Hammer and Spears (2016).

The potential efficacy of food markets is likely to operate primarily through dietary intake, as shown for Ethiopia by Abay and Hirvonen (2016). As shown by Puentes et al. (2016), total intake, especially of nutrient-rich foods, can have a major impact on child growth. In this study, we focus on whether households have access to food from elsewhere than their own farm production, by using their district’s share of total food consumption that is either purchased or received in-kind, as opposed to consumed on-farm. We employ district fixed effects to control for other time-invariant district characteristics, thereby isolating the specific effect of being in districts where households are more reliant on local conditions and unable to use markets to improve dietary quality, as in the study by Sibhatu et al. (2015).

### 2.5 Identification strategy and data sources

Our study uses a two-stage difference-in-differences design, comparing the association between child height and earlier agroclimatic conditions among children with different birth exposure, in households with and without toilets, and in districts with low and high food market use. The first set of differences exploits a natural experiment in exposure to different levels of NDVI at each stage of early development, while the second splits the sample into two groups to isolate the protective effects of sanitation and food markets. This strategy relies on combining three distinct kinds of information: NDHS data on child height and household characteristics, NDVI data on agroclimatic conditions, and NLSS data on agricultural commercialization. With these datasets, we are able to address a number of potential threats to identification: first, showing that the month of conception is uncorrelated with maternal and household socioeconomic status or other characteristics; second, using fixed effects and statistical controls to narrow the parallel-trends assumption implicit in our method; and third, performing a set of placebo regressions to demonstrate that results are not an artifact of the method. In all regressions, we control for observables known to correlate with maternal health and child size, such as parental education, maternal body mass index (BMI), household wealth, and altitude (Wehby et al., 2010).

The NDHS is a comprehensive, nationally representative survey typically conducted every five years to gather data on population and health. The NDHS is carried out under the Ministry of Health and Population and conducted by New ERA as part of the worldwide DHS program. The DHS uses standardized questionnaires and fieldwork to allow comparisons across years in demographics and health and nutrition–related variables. This paper uses two recent rounds of the NDHS conducted in 2006 (February–August) and 2011 (February–June). Both survey rounds use two-stage, stratified sampling, including households in all 75 districts across all ecological zones and development regions. The 2006 survey uses the 2001 population census for a sampling frame, while the 2011 survey uses an updated 2001 population census for a sampling frame that accounts for population growth and internal and external migration. Anthropometrics for children under five years of age were collected from 5237 and 2335 children in 2006 and 2011, respectively (Nepal MoHP, 2007, Nepal MoHP, 2012).

The NDVI is a measure of vegetation density resulting from the interaction among rainfall, temperature, and soil fertility over time. Green vegetation, owing to the presence of chlorophyll, absorbs red (visible) light and reflects near infrared light, while sparse vegetation reflects more red light and less near infrared light. NDVI values are measured as NDVI = NIR − RED/NIR + RED. Here, the numerator denotes the normalized difference between red and near infrared light bands, and the denominator is the sum of red and near infrared light bands (Weier and Herring, 2000). Those data are obtained from NASA satellite remote sensors using a Moderate Resolution Imaging Spectroradiometer (MODIS) Climate Modeling Grid (CMG), which provides data at 5 km resolution (Shively et al., 2015). The dataset includes monthly NDVI values for 12 years (February 2000–May 2012) for each child’s location of birth, corresponding with 260 and 289 clusters in 2006 and 2011 NDHS, respectively (Nepal MoHP, 2007, Nepal MoHP, 2012).

The full distributions of NDVI levels for each month are shown in Appendix A. This variable provides an attractive measure of green biomass and leaf area (Thenkabail et al., 2009), which in turn depend on available moisture, temperature, and soil fertility as well as human intervention in response to those underlying conditions (Laidler et al., 2008). NDVI is also influenced by factors other than plant growth, such as cloud cover and ground conditions. Although NDVI is not a simple index of climatic conditions or crop production (Shively et al., 2015), it does provide a powerful measure of trends and fluctuations in various agroclimatic conditions that could affect child development.

The NLSS is a comprehensive national, multitopic household survey conducted by the Nepal Central Bureau of Statistics (CBS), using survey methods developed and promoted by the World Bank. The NLSS uses multistage and stratified sampling with the Population Census 2001 of Nepal as a sample frame basis. Data collection spans an entire year, unlike the NDHS, in order to capture the effects of seasonality. The purpose of the NLSS is to assess changes in the population’s living standards using a combination of panel data and cross-sectional data for a given survey round. A wide range of topics covered by the NLSS includes poverty and access to finances, health, education, agriculture and rural development, and labor markets (Nepal CBS, 2004, Nepal CBS, 2011). This paper, however, uses only agriculture and rural development data, which include a range of information on food consumption, food production, and related expenses. In order to align the time frames between NLSS and NDHS data, the NLSS II 2003–2004 is merged with the NDHS 2006, and the NLSS III 2010–2011 is merged with the NDHS 2011 (Shively et al., 2015).

## 3 Data and results

Our main outcome variable is each child’s height-for-age *z* score (HAZ), defined as the gap between that child’s measured height and the median height of a healthy population at each age and sex, expressed in terms of standard deviations (SDs) of the healthy population (WHO and UNICEF, 2009). Children are classified as stunted when their HAZ is two or more SDs below the median, but here we focus on HAZ scores as a continuous variable to capture variation at every level of attained height. In addition, although the NDHS enumerators measured the length or height of all children under five years of age, here we focus on heights attained between the child’s first and fifth birthdays, as a function of agroclimatic conditions experienced both in utero and during the child’s first year after birth. Control variables include the child’s age in months at the time of measurement, total number of siblings ever born, maternal age, maternal education and BMI, household wealth, altitude and region, urban residence, and survey round.

### 3.1 Descriptive statistics

Table 1 shows means and SDs for nutritional outcomes and control variables in addition to other variables of interest. All data are from the NDHS 2006 and 2011, except for district-level data on food market participation and distance to market centers, which are obtained from the two waves of the NLSS. Because later regressions will be performed on data split by the sex of the child, we show the pooled dataset of all children aged 12–59 months (*N* = 6127), and the subsamples of boys (*n* = 3129) and girls (*n* = 2998).

The summary statistics in Table 1 show that the two subsamples are balanced in all regards, except that the male subsample is larger and the females have more siblings, which is consistent with sex-selective stopping rules by which parents seek additional children until they reach their desired number of boys (Bongaarts, 2013).

**Fig. 1:**
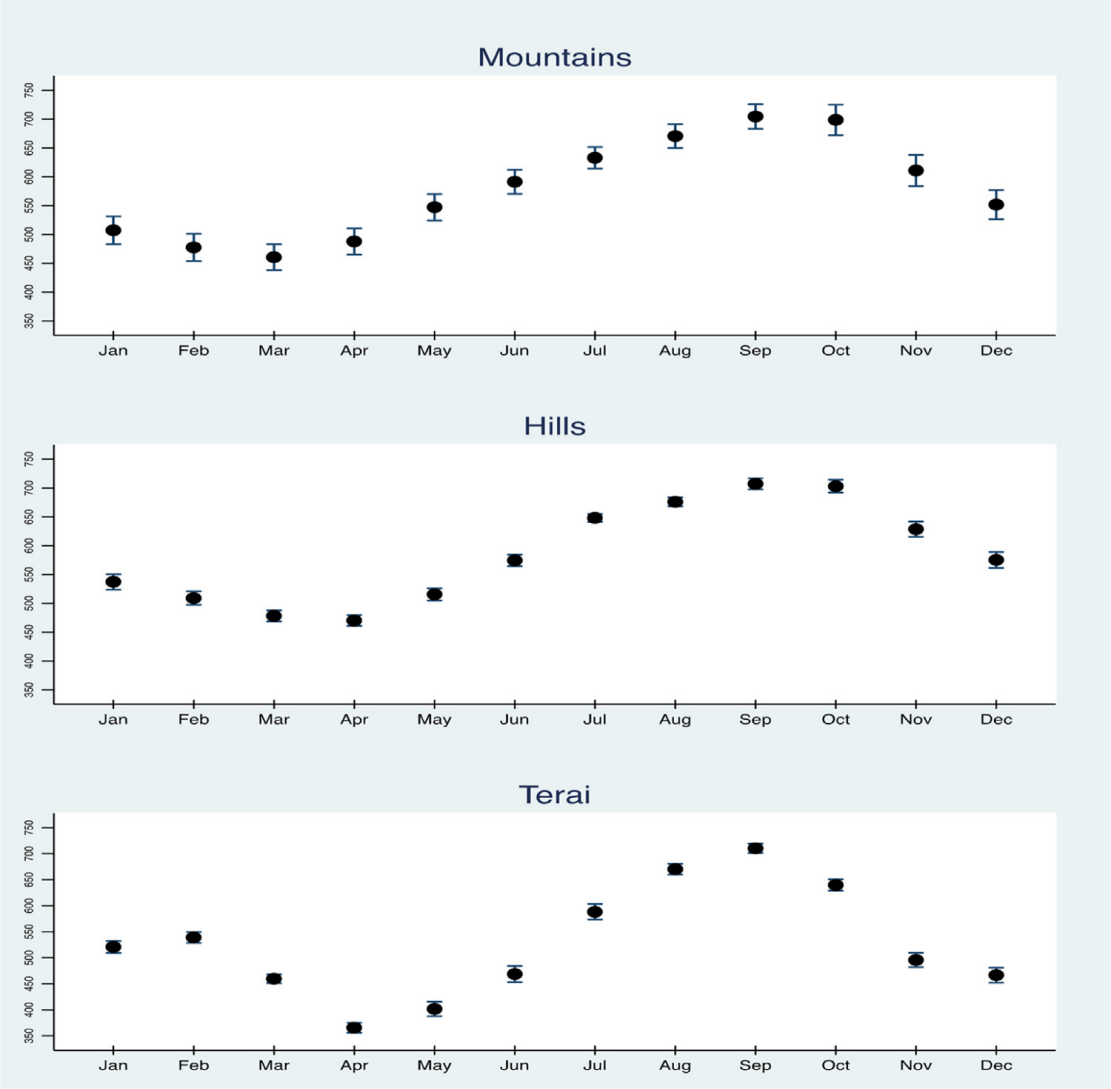
Month-to-month variation in mean NDVI at surveyed household locations, by region. Points shown are the mean Normalized Difference Vegetation Index (NDVI) in each month from 2000 through 2012 from grid cells around DHS survey clusters in each region, at 87 locations in the Mountains, 226 in the Hills, and 234 in the Terai. The height of each line represents a 95% confidence interval around that mean.

Turning to agroclimatic conditions, the NDVI levels to which each child is exposed at each stage of development depends on the month and location of birth. Fig. 1 shows the seasonal patterns of NDVI variation in Nepal’s three main regions. Vegetative cover generally peaks during August–October, with greater month-to-month variation occurring in the Terai region. There is greater uncertainty in calculating each month’s mean NDVI for the Mountain region, partly because of smaller sample size: only 87 of the 547 cluster locations for our two NDHS surveys were in the Mountains, while the rest were almost equally distributed between Hills and Terai.

### 3.2 Exploratory regressions

Our identification strategy relies on randomness in birth timing relative to variation in agroclimatic conditions around the home. Before proceeding, we test for possible selection effects, asking whether some kinds of mothers are more likely to conceive in certain months of the year. Seasonal patterns of conception could result from seasonal migration of family members, variation in natural fertility, or even deliberate pursuit of conception at more favorable times. Table 2 tests for selection patterns in a very general way, using a multinomial logit model of selection for each month of conception, nine months before the observed month of birth.

The results of Table 2 show 12 of 121 coefficients with statistical significance at the 5 percent level or higher. Half of these are the significant coefficients on altitude between February and August. During that period, families at higher altitudes are more likely to conceive a child than families at lower altitudes. Lower altitude places are relatively hot in the summer, so this result is consistent with Levitas et al. (2013) who found sperm counts and motility to be lower at warmer times and places. The remaining six coefficients which are significant at the 95 percent level have no clear pattern, confirming the absence of selection effects on the timing of conception in this context. These results contrast with the strong selection effects in the United States (Buckles and Hungerman, 2013) but are consistent with studies elsewhere, such as Taiwan (Fan et al., 2014), India (Lokshin and Radyakin, 2012), and Nepal itself (Panter-Brick, 1996).

Without selection by education or other parental characteristics for specific birth months, differences in attained height by month of birth are most likely the result of environmental exposure effects. To identify this purely seasonal pattern in how birth timing relates to attained heights, we employ ordinary least squares (OLS) regressions using the following base specification:

(1) *Y_i_* = β_0_ + β_1*m*_*birthmonth_im_* + *δ_i_Z_i_* + *u_i_*,

where *Y_i_* indicates the HAZ score of a child aged 12–59 months, *birthmonth_im_* is a vector of dummy variables equal to 1 when *m* = the birth month of that child and zero otherwise, and *Z_i_* is a vector of control variables at the child-, maternal-, household-, and district levels. District fixed effects are also included, and standard errors are clustered by birth year and district. To account for the effects of a child’s age on measured HAZ, as in Cummins (2015), we use two different specifications: age and age squared in columns 1, 3, and 5, and a linear year of birth term in columns 2, 4, and 6. The two types of age control yield a similar month of birth effects, so for the remaining tests, we use only the simple age in months and months-squared specification, and focus attention on the differences between boys and girls in regard to what birth timing might be relatively unfavorable.

Results in Table 3 reveal that the worst month for all children to be born is April, but boys are also disadvantaged by being born in June and September relative to the omitted month, January. As shown in Fig. 3 .1, April is one of the lowest-NDVI months, while September the highest. A few other months have some correlation with attained heights, and the magnitude of these seasonality effects are very large: for all children, being born in April has an effect size similar to 1.9 quintiles of wealth, and for boys, being born in September has an effect size similar to 2.4 quintiles of wealth.

### 3.3 Empirical methods

To investigate causal mechanisms and potential remedies for the effect of birth timing on attained height, we turn to variation in NDVI at each stage of child development during pregnancy and the first four three-month periods after birth. Our focus is on heterogeneity and effect modifiers, so we divide the sample into boys and girls to identify sex-specific vulnerability at each stage of early child development, and then test for the protective effects of sanitation and food markets by dividing the sample into households with or without toilets and districts with above- or below-median reliance on local production as opposed to food purchases or gifts. This split-sample approach, made possible by our large number of observations, permits variation among subsamples in all coefficients while avoiding a proliferation of collinear interaction terms.

In this design, exposure to climate variation is a natural experiment to which children are exposed under conditions that might or might not be protective. Testing for heterogeneity in treatment response can help identify causal mechanisms, target services to those most at risk, and help spread desirable effect modifiers. In this case, we seek to identify the protective effects of toilets and food markets against adverse “treatments” that cannot be experimentally assigned, but are experienced randomly by children in utero and during the first year after birth.

In each subsample, we employ OLS with the following base specification:

(2) *Y_i_* = β_0_ + β_1*t*_ NDVI*_it_* + *δ_i_Z_i_* + *u_i_*

Our notation is the same as for Eq. (1), except that the vector of NDVI*_it_* conditions is the average value of NDVI in each three-month period *t*, from the first trimester of pregnancy through the first year after birth when the child is aged 0–2 months, 3–5 months, 6–8 months, and 9–11 months. All *Z_i_* control variables are the same as in our exploratory regressions, first using both types of age controls, and then using only the more flexible quadratic approach to controlling for the child’s age at the time of measurement.

Our hypothesis is that the β_1*t*_ coefficients of significance in the whole sample (Table 4) become insignificant among households with toilets (Table 5) and in districts with more food market activity (Table 6). We conduct this difference-in-differences test using a split sample approach to gain maximum flexibility for the coefficients on each regressor. The test is powered by a large sample size achieved through merging two DHS rounds, and a large magnitude of the baseline β**_1_** effects which could be brought to zero by sanitation and food markets.

The results of Table 4 are similar using either the flexible specification in columns 1, 3, and 5, or the linear specifications in 2, 4 and 6. In both cases, higher NDVI in the second trimester of pregnancy is associated with greater attained heights, but only for boys. Girls have lower attained heights when NDVI is higher in the first three months after birth. Boys also have lower attained heights when NDVI is higher in months 3–5 after birth, but only in the less flexible functional form for age at measurement used in column 4.

The magnitudes of effects are quite large. Coefficients shown in Table 4 are scaled per 1000 points of NDVI, so based on our preferred specifications in columns 3 and 5, each 100-point change in NDVI experienced by boys in midgestation is associated with an 0.088 difference in HAZ—almost as large as the 0.107 difference associated with each quintile of household wealth. For girls, each 100-point change in NDVI experienced during the first three months after birth is associated with a 0.054 difference in HAZ, which is almost half of the 0.112 difference associated with each quintile of wealth. Coefficients on wealth and other control variables are similar in direction and significance for both boys and girls, except that the magnitude of coefficients is somewhat larger for boys, suggesting greater susceptibility to variables such as altitude and maternal age.

Turning to the effects of NDVI on households with and without toilets, from Table 5 we observe the predicted heterogeneity in effect sizes and significance. In households without toilets, fluctuations in NDVI significantly affect males only in utero (column 3) and significantly affect females only in the first three months after birth (column 5). Households with toilets are largely immune to the effects of NDVI fluctuations at any stage of early child development. Effect sizes for the subsample without toilets are larger than for the country as a whole: a 100-point change in NDVI during the second trimester of pregnancy affects boys’ heights as much as the equivalent of 1.2 quintiles of wealth, while a 100-point change in NDVI during the first three months after birth affects girls’ heights as much as the equivalent of 1.6 quintiles of wealth. However, those effects completely disappear in households with toilets.

The effects of NDVI on children’s height in districts with higher or lower levels of food market activity (shown in Table 6) reveal a murkier picture. In the low-market-use districts, girls are susceptible to NDVI fluctuations only in their first three months after birth, and boys are susceptible to NDVI fluctuation in their second trimester of gestation, but these subsamples also show a significant correlation with NDVI during the period of complementary feeding (six to eight months of age). This particular correlation holds true for boys in districts with low food market use, and for girls in districts with high food market use. This could be a spurious correlation arising by chance in this sample, in part because these two periods in a child’s life occur in the same season in successive years, so the two NDVIs are highly collinear. Effect sizes for the main results are roughly similar here as in the previous table. For boys in districts with low food market participation, a 100-point change in NDVI in midgestation has an effect size similar to 1.5 quintiles of wealth; for girls, a 100-point change in NDVI in the three months after birth has a smaller effect, similar to about 0.5 quintiles of wealth.

### 3.4 Robustness checks

To test the robustness of our results, we look for artifacts of the method by checking whether our regressions generate more statistically significant results than would arise by chance. These placebo regressions use the same data as our main results in Table 5, Table 6, with dependent variables that were determined long before the NDVI fluctuations occurred, so no causal effect is possible. These variables consist primarily of maternal characteristics (columns 1–6) plus household wealth and urban or rural location (columns 7 and 8). The base rate of entirely spurious placebo effects is proportional to *p-*values; for example, one tenth of the effects appears significant at a *p-*value of 10 percent. Table 7 reveals that, among the 7 × 8 = 56 distinct placebo “treatments” tested on each age-group, only five effects arise with a *p-*value < 0.1, four effects with a *p*-value < 0.05, and one with a *p-*value < 0.01.

The number and pattern of significant coefficients in Table 7 correspond to the frequency of significant correlations that would be expected to occur by chance alone, and there is no time-related pattern to these correlations, as each occurs at a different stage of child development. Because our placebo regression tests used the same data and model structure as Table 5, Table 6, the clear pattern that we observe for NDVI treatment effects on child height is very likely to be a robust result of causal mechanisms.

## 4 Conclusions

The objective of this study is to identify the influence of early-life agroclimatic conditions on children’s attained heights in Nepal, and to test for the protective effects of household sanitation and food markets. In so doing, we find clear heterogeneity in vulnerability to changes in NDVI: boys are most affected by agroclimatic conditions during their second trimester of gestation, whereas girls are most vulnerable in the three months after birth. These findings are consistent with biomedical studies of sex-specific fetal development and socioeconomic studies of gender bias in child care. Both kinds of vulnerability are eliminated in households with toilets, and greatly reduced in districts that have more active use of food markets.

The magnitude of fluctuations against which sanitation and food markets are protective is very large: on average, in households without toilets, for boys each 100-point variation in NDVI during the second trimester of gestation is associated with as much difference in attained height as conferred by 1.2 quintiles of household wealth, and for girls that same variation during the first three months after birth is associated with a difference in height equivalent to 1.6 quintiles of household wealth. The average seasonal change in NDVI from peak to trough in Nepal during the 2000–2011 period is approximately 200 points in the Mountains and Hills regions and 300 points in the Terai (lowland) region. The resulting difference in attained height between the most and least favorable times of year depends on location but is similar to the differences associated with about one to three quintiles of wealth. To test the internal validity of these findings, we conducted a number of placebo regressions that revealed no significant artifactual correlations that could have resulted from selection effects in birth timing.

Heterogeneity in response to agroclimatic conditions provides important clues as to the causal mechanisms involved, and valuable guidance regarding the targeting of interventions. Our results are consistent with the premise that household sanitation protects children against fecal–oral transmission of disease, while food purchases made possible by more robust food markets protect children against fluctuations in local food production. Thus, policy interventions to promote sanitation and strengthen food markets could help protect children against adverse conditions in the future, helping them to grow up healthy despite the effects of climate change. Our research design exploited random exposure to varying agroclimatic circumstances in relation to birth timing. To detect selection effects, we test for the effects of socioeconomic status on month of birth and found no correlations. We also use a series of placebo regressions to test whether our research design generates spurious correlations, and find only the expected base rate of noncausal statistical significance. These empirical tests increase confidence in the robustness of our results for the Nepali context.

The main limitation of our study concerns the protective effects of sanitation and food markets, as household toilets and market activity were not randomly assigned. Our regressions control for observable influences on child height, such as household wealth, parental education, maternal BMI, and altitude as well as district fixed effects. Violations of the parallel-trends assumption behind our difference-in-differences design would involve other factors that make households less vulnerable to agroclimatic conditions, such as cultural differences. For example, perhaps households with toilets and in districts with stronger food markets have access to more effective social insurance than the average household with similar levels of observable control variables. While the large protective effects found in our data are plausibly explained by differences in fecal–oral disease transmission and food consumption smoothing, cultural differences or other unobserved factors cannot be ruled out without conducting large-scale, randomized trials.

Future work could extend our results using other natural experiments in observational data, perhaps over longer periods and more countries, or refining the measurement of agroclimatic conditions and the timing of exposure. This study confirms that exploiting natural experiments can be of great value in identifying the periods in utero and during infancy when child development is most at risk, and extends previous results to reveal the socioeconomic conditions that are most able to protect children against those risks. These results point to the power of investments in maternal health during pregnancy to protect boys, and in immediate postnatal care to protect girls, as well as the value of sanitation and food markets for building resilience against variation in agroclimatic conditions.

## Funding

This project was supported by the United States Agency for International Development through the Feed the Future Innovation Lab for Nutrition [grant number AID-O-AA-1-1-00005, for work by WAM, PM and GES], and by the Bill & Melinda Gates Foundation through the International Food Policy Research Institute [project number 301052.001.001.515.01.01, for SAB and WAM].

## Figures and Tables

**Table 1:** Summary statistics for all outcome and control variables.

Variable	(1) All observations	(2) Male	(3) Female	(4) Male vs. female
	(*N* = 6127)	(*n* = 3129)	(*n* = 2998)	(*p*-value)
Nutritional status outcomes				
HAZ	−2.11 (1.25)	−2.10 (1.24)	−2.12 (1.26)	0.6363
Mother’s BMI (kg/m^2^)	20.55 (2.74)	20.54 (2.77)	20.57 (2.71)	0.7546
Child characteristics				
Female	48.9%	n.a.	n.a.	n.a.
Child age (months)	35.65 (13.75)	35.89 (13.66)	35.41 (13.85)	0.1736
Number of siblings ever born	2.11 (1.96)	2.04 (1.93)	2.17 (1.99)	**0.0088**
Maternal characteristics				
Age (years)	27.42 (6.06)	27.37 (5.90)	27.46 (6.22)	0.5811
Primary education completed	18.0%	18.1%	17.9%	0.857
Secondary education completed	21.2%	21.4%	21.1%	0.822
Tertiary education completed	3.3%	3.3%	3.2%	0.846
Household characteristics				
Wealth (quintile)	2.66 (1.42)	2.67 (1.43)	2.65 (1.42)	0.6013
Has toilet	45.7%	46.6%	44.7%	0.146
District-level characteristics				
Food market participation (share of food consumption purchased or donated)	0.52 (0.20)	0.52 (0.20)	0.52 (0.20)	0.6698
Urbanization (% rural)	78.2%	78.3%	78.1%	0.863
Altitude (average, m)	833.42 (732.18)	846.86 (734.87)	819.38 (729.23)	0.1420

*Note*: Summary statistics pertain to all observations included in regressions (every measured child aged 12–59 months). Data presented in columns 1–3 are means (SD) or percentages. Column 4 shows *p-*values of comparisons between male and female children using either the chi-square or independent sample *t*-test, as appropriate, in bold if less than 1%. HAZ = height-for-age *z*-score.

**Table 2:** Timing of conception and maternal and household characteristics, by month.

	(1)	(2)	(3)	(4)	(5)	(6)	(7)	(8)	(9)	(10)	(11)
Variable	January	February	March	April	May	June	July	August	September	October	November
Maternal age (log)	0.029	−0.111	−0.357	−0.348	0.574	0.239	0.217	−0.157	0.272	0.034	0.487
	(0.42)	(0.44)	(0.44)	(0.44)	(0.43)	(0.43)	(0.46)	(0.46)	(0.42)	(0.42)	(0.41)
Maternal primary education	0.133	0.222	0.047	0.410**	0.262	0.240	0.456**	0.089	0.442**	0.146	0.468***
	(0.18)	(0.18)	(0.18)	(0.17)	(0.18)	(0.18)	(0.18)	(0.19)	(0.17)	(0.18)	(0.17)
Maternal secondary education	0.077	0.205	0.124	0.125	0.048	0.037	0.191	−0.031	0.146	0.192	0.014
	(0.18)	(0.19)	(0.18)	(0.19)	(0.18)	(0.19)	(0.20)	(0.20)	(0.18)	(0.18)	(0.18)
Maternal tertiary education	0.157	0.155	0.497	0.113	0.392	0.105	0.577	0.224	0.473	0.095	0.540
	(0.39)	(0.41)	(0.38)	(0.40)	(0.38)	(0.42)	(0.41)	(0.41)	(0.38)	(0.40)	(0.36)
Maternal BMI (kg/m^2^)	−0.042*	−0.013	−0.002	−0.045*	−0.054**	−0.023	−0.009	−0.006	0.007	−0.022	0.003
	(0.02)	(0.02)	(0.02)	(0.02)	(0.02)	(0.02)	(0.02)	(0.03)	(0.02)	(0.02)	(0.02)
Total children ever born	−0.038	0.004	0.012	0.037	−0.040	−0.010	0.043	0.033	0.009	0.031	−0.007
	(0.05)	(0.05)	(0.05)	(0.05)	(0.05)	(0.05)	(0.05)	(0.05)	(0.05)	(0.05)	(0.05)
Wealth (quintile)	0.003	−0.048	–0.021	0.051	0.010	−0.004	−0.057	−0.005	−0.023	−0.008	0.015
	(0.05)	(0.06)	(0.05)	(0.06)	(0.05)	(0.06)	(0.06)	(0.06)	(0.05)	(0.05)	(0.05)
Altitude	0.042	0.247**	0.174*	0.272***	0.235**	0.265**	0.234**	0.367***	0.187*	0.179*	0.123
	(0.10)	(0.10)	(0.10)	(0.10)	(0.10)	(0.10)	(0.11)	(0.11)	(0.10)	(0.10)	(0.10)
2011 DHS observation	−0.015	0.279**	−0.063	0.032	0.029	−0.104	0.015	0.195	0.128	−0.009	0.001
	(0.14)	(0.13)	(0.14)	(0.14)	(0.13)	(0.14)	(0.14)	(0.14)	(0.13)	(0.13)	(0.13)
Hill	0.510**	0.312	0.322*	0.120	0.189	0.084	0.194	0.259	0.181	0.428**	0.151
	(0.21)	(0.19)	(0.19)	(0.19)	(0.19)	(0.19)	(0.19)	(0.19)	(0.19)	(0.19)	(0.19)
Terai	0.425	0.549*	0.373	0.383	0.394	0.358	0.306	0.424	0.236	0.523*	0.263
	(0.33)	(0.32)	(0.31)	(0.31)	(0.31)	(0.32)	(0.35)	(0.33)	(0.31)	(0.31)	(0.31)
Constant	0.054	−1.435	−0.259	−0.252	−2.504*	−2.284	−2.584	−2.338	−2.570*	−1.355	−2.790*
	(1.45)	(1.51)	(1.54)	(1.51)	(1.51)	(1.52)	(1.66)	(1.63)	(1.51)	(1.46)	(1.44)
Observations (*N*)	6127	6127	6127	6127	6127	6127	6127	6127	6127	6127	6127

*Note*: Unit of observation = individual child aged 12–59 months. Results shown are a multinomial logit model of selection for each month of conception, which is inferred to be nine months before the observed month of birth (for example, April births imply conception in July). Coefficients are relative to the omitted month, December. Robust standard errors are in parentheses. 2011 DHS observation indicates dummy variable = 1 if the DHS survey was conducted in 2011. Hill and Terai dummy variables indicate likelihood compared with the Mountain region. BMI = body mass index; DHS = Nepal Demographic and Health Surveys; ***p < 0.01, **p < 0.05, *p < 0.1.

**Table 3:** Child height and month of birth by sex, with alternative controls for age.

	(1)	(2)	(3)	(4)	(5)	(6)
Variable	Both sexes, age in months	Both sexes, age in years	Males, age in months	Males, age in years	Females, age in months	Females, age in years
February	−0.010	0.001	−0.042	−0.032	0.020	0.034
	(0.08)	(0.08)	(0.12)	(0.12)	(0.09)	(0.09)
March	−0.077	−0.054	−0.085	−0.067	−0.057	−0.027
	(0.09)	(0.08)	(0.13)	(0.13)	(0.06)	(0.05)
April	−0.198***	−0.321***	–0.188***	−0.279***	−0.213**	−0.349***
	(0.06)	(0.07)	(0.06)	(0.09)	(0.09)	(0.10)
May	−0.036	−0.125	−0.139	–0.208*	0.078	−0.026
	(0.07)	(0.10)	(0.10)	(0.12)	(0.08)	(0.12)
June	−0.143**	−0.211**	−0.235**	−0.287***	−0.059	−0.137
	(0.07)	(0.08)	(0.10)	(0.11)	(0.08)	(0.09)
July	−0.108	−0.161*	−0.091	−0.127	−0.111	−0.175
	(0.08)	(0.09)	(0.10)	(0.10)	(0.09)	(0.12)
August	−0.115	−0.150	−0.144	−0.159	−0.087	−0.142
	(0.09)	(0.11)	(0.10)	(0.12)	(0.12)	(0.13)
September	−0.127	−0.159	−0.277***	−0.288***	0.030	−0.017
	(0.09)	(0.11)	(0.09)	(0.11)	(0.13)	(0.15)
October	−0.090	−0.112	−0.197*	−0.212*	0.002	−0.027
	(0.11)	(0.11)	(0.11)	(0.12)	(0.14)	(0.15)
November	−0.059	−0.076	0.013	0.002	−0.169	−0.193*
	(0.10)	(0.10)	(0.10)	(0.10)	(0.11)	(0.11)
December	−0.099	−0.107	−0.182	−0.181	−0.011	−0.022
	(0.10)	(0.10)	(0.13)	(0.13)	(0.10)	(0.10)
Female	−0.017	−0.013	n.a.	n.a.	n.a.	n.a.
	(0.04)	(0.04)				
Age (months)	−0.067***	n.a.	−0.061***	n.a.	−0.078***	n.a.
	(0.01)		(0.01)		(0.01)	
Age squared (months)	0.001***	n.a.	0.001***	n.a.	0.001***	n.a.
	(0.00)		(0.00)		(0.00)	
Number of siblings ever born	−0.073***	−0.073***	−0.080***	−0.081***	−0.061***	−0.061***
	(0.01)	(0.01)	(0.02)	(0.02)	(0.01)	(0.01)
Maternal age (log)	0.410***	0.403***	0.535***	0.529***	0.298*	0.299**
	(0.11)	(0.11)	(0.16)	(0.16)	(0.16)	(0.15)
Maternal primary education	0.064*	0.064**	0.093*	0.093*	0.056	0.060
	(0.03)	(0.03)	(0.05)	(0.05)	(0.06)	(0.06)
Maternal secondary education	0.305***	0.310***	0.336***	0.342***	0.289***	0.292***
	(0.06)	(0.06)	(0.06)	(0.06)	(0.08)	(0.08)
Maternal tertiary education	0.664***	0.672***	0.610***	0.618***	0.750***	0.759***
	(0.09)	(0.09)	(0.07)	(0.06)	(0.15)	(0.14)
Maternal BMI (kg/m^2^)	0.041***	0.041***	0.046***	0.044***	0.037***	0.037***
	(0.01)	(0.01)	(0.01)	(0.01)	(0.01)	(0.01)
Wealth (quintile)	0.107***	0.107***	0.107***	0.109***	0.111***	0.109***
	(0.02)	(0.02)	(0.02)	(0.02)	(0.02)	(0.02)
Altitude (log)	−0.266***	−0.264***	−0.299***	−0.301***	−0.230**	−0.224**
	(0.08)	(0.08)	(0.08)	(0.08)	(0.09)	(0.09)
2011 DHS observation	0.141***	−0.642***	0.112**	−0.387***	0.187***	−0.887***
	(0.04)	(0.10)	(0.06)	(0.11)	(0.05)	(0.17)
Hill	−0.104	−0.118	0.048	0.043	−0.189	−0.202
	(.)	(.)	(.)	(.)	(.)	(.)
Terai	−0.277*	−0.232	−0.358**	−0.310**	−0.194	−0.159
	(0.15)	(0.15)	(0.16)	(0.15)	(0.26)	(0.25)
Urban location	0.022	0.028	−0.024	−0.022	0.052	0.061
	(0.06)	(0.06)	(0.05)	(0.05)	(0.07)	(0.07)
Year of birth	n.a.	0.212***	n.a.	0.156***	n.a.	0.257***
		(0.04)		(0.05)		(0.04)
Constant	−1.392**	−427.182***	−1.715***	−314.317***	−1.160	−516.367***
	(0.55)	(89.35)	(0.63)	(96.70)	(0.76)	(81.48)
Observations (*n*)	6127	6127	3129	3129	2998	2998
*R*-squared	0.187	0.187	0.197	0.199	0.209	0.209

*Note*: Unit of observation = individual child aged 12–59 months. Robust standard errors in parentheses, clustered at the birth year and district levels. All regressions include fixed effects for district (*n* = 75). Omitted month is January, and omitted region is Mountains; standard error for Hill districts is not estimated due to multicollinearity. 2011 DHS observation = 1 if the DHS survey was conducted in 2011. BMI = body mass index; DHS = Nepal Demographic and Health Surveys; n.a. = not applicable; ***p < 0.01, **p < 0.05, *p < 0.1.

**Table 4:** Child height and NDVI before and after birth by sex, with alternative controls for age.

	(1)	(2)	(3)	(4)	(5)	(6)
Variable	Both sexes, age in months	Both sexes, age in years	Males, age in months	Males, age in years	Females, age in months	Females, age in years
First trimester	−0.102	0.034	−0.201	0.110	−0.074	−0.063
	(0.28)	(0.37)	(0.27)	(0.33)	(0.27)	(0.37)
Second trimester	0.395**	0.445**	0.879***	1.016***	−0.225	−0.236
	(0.16)	(0.20)	(0.29)	(0.30)	(0.36)	(0.42)
Third trimester	0.358	0.352	0.451	0.432	0.297	0.337
	(0.39)	(0.42)	(0.39)	(0.45)	(0.49)	(0.52)
0–2 months of age	−0.313*	−0.308	−0.086	−0.041	−0.544**	−0.575*
	(0.17)	(0.20)	(0.29)	(0.28)	(0.26)	(0.31)
3–5 months of age	−0.123	−0.422	−0.164	−0.608***	−0.044	−0.247
	(0.31)	(0.34)	(0.24)	(0.23)	(0.36)	(0.38)
6–8 months of age	−0.309*	−0.262*	−0.335	−0.408	−0.123	0.030
	(0.16)	(0.15)	(0.34)	(0.33)	(0.31)	(0.37)
9–11 months of age	−0.238	−0.123	−0.366	−0.250	−0.179	−0.099
	(0.36)	(0.37)	(0.49)	(0.50)	(0.41)	(0.42)
Female sex	−0.016	−0.012	n.a.	n.a.	n.a.	n.a.
	(0.04)	(0.04)				
Age (months)	−0.068***	n.a.	−0.064***	n.a.	−0.076***	n.a.
	(0.01)		(0.01)		(0.01)	
Age squared	0.001***	n.a.	0.001***	n.a.	0.001***	n.a.
	(0.00)		(0.00)		(0.00)	
Number of siblings ever born	−0.073***	−0.073***	−0.080***	−0.079***	−0.064***	−0.065***
	(0.01)	(0.01)	(0.02)	(0.02)	(0.01)	(0.01)
Maternal age (log)	0.413***	0.399***	0.550***	0.532***	0.323**	0.316**
	(0.11)	(0.11)	(0.15)	(0.15)	(0.16)	(0.16)
Maternal primary education	0.066**	0.066**	0.105**	0.105**	0.055	0.058
	(0.03)	(0.03)	(0.05)	(0.05)	(0.06)	(0.06)
Maternal secondary education	0.305***	0.310***	0.341***	0.348***	0.284***	0.286***
	(0.06)	(0.06)	(0.07)	(0.07)	(0.08)	(0.08)
Maternal tertiary education	0.664***	0.670***	0.607***	0.612***	0.743***	0.750***
	(0.09)	(0.09)	(0.07)	(0.07)	(0.15)	(0.14)
Maternal BMI (kg/m^2^)	0.040***	0.040***	0.046***	0.044***	0.037***	0.037***
	(0.01)	(0.01)	(0.01)	(0.01)	(0.01)	(0.01)
Wealth (quintile)	0.108***	0.108***	0.107***	0.110***	0.112***	0.110***
	(0.02)	(0.02)	(0.02)	(0.02)	(0.02)	(0.02)
Altitude (log)	−0.254***	−0.252***	−0.304***	−0.308***	−0.200**	−0.194*
	(0.08)	(0.08)	(0.09)	(0.09)	(0.10)	(0.10)
2011 DHS	0.134***	−0.558***	0.106*	−0.324***	0.174***	−0.803***
	(0.04)	(0.11)	(0.06)	(0.11)	(0.05)	(0.17)
Hill	−0.129	−0.143	0.053	0.054	−0.267	−0.283
	(.)	(.)	(.)	(.)	(.)	(.)
Terai	−0.290**	−0.247*	−0.357*	−0.305*	−0.235	−0.201
	(0.15)	(0.14)	(0.18)	(0.18)	(0.24)	(0.22)
Urban location	0.017	0.022	−0.025	−0.026	0.037	0.047
	(0.06)	(0.06)	(0.06)	(0.06)	(0.07)	(0.07)
Year of birth	n.a.	0.141***	n.a.	0.050	n.a.	0.230***
		(0.05)		(0.05)		(0.07)
Constant	−1.340**	−285.369***	−1.930***	−103.063	−0.923	−463.169***
	(0.55)	(106.24)	(0.66)	(101.59)	(0.71)	(136.52)
Observations (*n*)	6127	6127	3129	3129	2998	2998
*R*-squared	0.187	0.186	0.196	0.197	0.207	0.205

*Note*: Unit of observation = individual child aged 12–59 months. Robust standard errors in parentheses, clustered at the birth year and district levels. All regressions include fixed effects for district (*n* = 75). Omitted month is January and omitted region is Mountains; standard error for Hill districts is not estimated due to multicollinearity. 2011 DHS observation = 1 if the DHS survey was conducted in 2011. Rows 1–7 show NDVI in each trimester of pregnancy and three-month period of the child’s first year, scaled to show difference in HAZ score per 1000-point difference in NDVI. BMI = body mass index; DHS = Nepal Demographic and Health Surveys; n.a. = not applicable; ***p < 0.01, **p < 0.05, *p < 0.1.

**Table 5:** Child height and NDVI before and after birth by sex and presence of toilet in household.

Variable	(1)	(2)	(3)	(4)	(5)	(6)
	Both sexes	Both sexes	Male	Male	Female	Female
	no toilet	has toilet	no toilet	has toilet	no toilet	has toilet
First trimester	−0.071	−0.070	−0.397	0.109	0.100	−0.231
	(0.34)	(0.35)	(0.58)	(0.48)	(0.43)	(0.21)
Second trimester	0.522***	0.282*	1.016***	0.586	−0.172	−0.115
	(0.16)	(0.14)	(0.38)	(0.61)	(0.43)	(0.31)
Third trimester	0.470	0.168	0.522**	0.275	0.392	0.133
	(0.38)	(0.56)	(0.22)	(0.71)	(0.59)	(0.65)
0–2 months of age	−0.445*	−0.010	−0.041	0.003	−0.869***	−0.054
	(0.25)	(0.20)	(0.37)	(0.35)	(0.31)	(0.29)
3–5 months of age	−0.179	−0.066	−0.137	−0.201	−0.105	−0.100
	(0.28)	(0.34)	(0.34)	(0.53)	(0.52)	(0.26)
6–8 months of age	−0.568*	0.003	−0.691	0.183	−0.230	−0.203
	(0.30)	(0.31)	(0.43)	(0.60)	(0.61)	(0.42)
9–11 months of age	−0.299	−0.167	−0.401	−0.209	−0.237	−0.232
	(0.36)	(0.48)	(0.47)	(0.66)	(0.51)	(0.64)
Female sex	−0.086*	0.061	n.a.	n.a.	n.a.	n.a.
	(0.05)	(0.06)				
Age (months)	−0.061***	−0.079***	−0.059***	−0.070***	−0.068***	−0.092***
	(0.01)	(0.01)	(0.02)	(0.01)	(0.01)	(0.01)
Age squared (months)	0.001***	0.001***	0.001***	0.001***	0.001***	0.001***
	(0.00)	(0.00)	(0.00)	(0.00)	(0.00)	(0.00)
Number of siblings ever born	−0.068***	−0.086***	−0.082***	−0.079***	−0.055***	−0.089***
	(0.01)	(0.01)	(0.02)	(0.03)	(0.01)	(0.03)
Maternal age (log)	0.466***	0.390**	0.589**	0.587**	0.362*	0.288
	(0.15)	(0.17)	(0.27)	(0.25)	(0.19)	(0.20)
Maternal primary education	0.100**	0.025	0.121**	0.096	0.084	0.005
	(0.04)	(0.07)	(0.06)	(0.10)	(0.06)	(0.10)
Maternal secondary education	0.249***	0.285***	0.323***	0.355***	0.221**	0.227**
	(0.08)	(0.07)	(0.09)	(0.09)	(0.10)	(0.11)
Maternal tertiary education	1.190***	0.595***	1.336***	0.575***	1.071***	0.648***
	(0.22)	(0.11)	(0.16)	(0.12)	(0.39)	(0.16)
Maternal BMI (kg/m^2^)	0.033***	0.044***	0.039***	0.051***	0.030**	0.041***
	(0.01)	(0.01)	(0.01)	(0.01)	(0.01)	(0.01)
Wealth (quintile)	0.067***	0.095***	0.082***	0.090***	0.056**	0.095***
	(0.02)	(0.02)	(0.02)	(0.03)	(0.02)	(0.03)
Altitude	−0.135	−0.264***	−0.170	−0.314***	−0.118	−0.207
	(0.08)	(0.07)	(0.10)	(0.09)	(0.12)	(0.14)
2011 DHS observation	0.215***	0.077*	0.181**	0.046	0.273***	0.096
	(0.06)	(0.04)	(0.08)	(0.07)	(0.09)	(0.06)
Hill	−0.093	−0.199	−0.216	0.213	0.036	−0.496
	(.)	(.)	(.)	(.)	(0.09)	(.)
Terai	0.128***	−0.380**	−0.043	−0.354	0.207	−0.416
	(0.04)	(0.16)	(.)	(0.28)	(0.23)	(0.38)
Urban location	−0.081	0.091	−0.058	0.038	−0.121	0.145***
	(0.09)	(0.07)	(0.12)	(0.09)	(0.10)	(0.05)
Constant	−2.209***	−1.223	−2.712***	−2.282*	−1.669**	−0.283
	(0.45)	(0.80)	(0.78)	(1.20)	(0.77)	(1.31)
Observations (*n*)	3329	2797	1672	1457	1657	1340
*R*-squared	0.134	0.221	0.169	0.230	0.143	0.272

*Note*: Unit of observation = individual child aged 12–59 months. Robust standard errors in parentheses, clustered at the birth year and district levels. All regressions include fixed effects for district (*n* = 75). Omitted month is January and omitted region is Mountains; standard error for Hill and Terai districts in some regressions is not estimated due to multicollinearity. 2011 DHS observation = 1 if the DHS survey was conducted in 2011. Rows 1–7 show NDVI in each trimester of pregnancy and three-month period of the child’s first year, scaled to show difference in HAZ score per 1000-point difference in NDVI. BMI = body mass index; DHS = Nepal Demographic and Health Surveys; n.a. = not applicable; ***p < 0.01, **p < 0.05, *p < 0.1.

**Table 6:** Child height and NDVI before and after birth by sex and use of food markets.

	(1)	(2)	(3)	(4)	(5)	(6)
	Both sexes	Both sexes	Males	Males	Females	Females
	low-market use	high-market use	low-market use	high-market use	low-market use	high-market use
First trimester	0.054	−0.292	−0.170	−0.231	0.151	−0.337
	(0.33)	(0.31)	(0.55)	(0.53)	(0.25)	(0.41)
Second trimester	0.411*	0.399	1.346***	0.416	−0.610	0.395
	(0.22)	(0.39)	(0.14)	(0.54)	(0.45)	(0.54)
Third trimester	0.178	0.446	0.420	0.314	−0.073	0.381
	(0.36)	(0.60)	(0.61)	(0.56)	(0.23)	(0.86)
0–2 months of age	−0.211	−0.520	0.137	−0.353	−0.650*	−0.571
	(0.25)	(0.34)	(0.23)	(0.46)	(0.34)	(0.49)
3–5 months of age	−0.379	0.048	−0.231	−0.073	−0.485	0.178
	(0.42)	(0.29)	(0.50)	(0.54)	(0.38)	(0.44)
6–8 months of age	−0.468**	−0.254	−1.088***	0.254	0.260	−0.801***
	(0.22)	(0.34)	(0.28)	(0.54)	(0.48)	(0.29)
9–11 months of age	−0.406	−0.097	−0.501	−0.105	−0.292	−0.041
	(0.31)	(0.54)	(0.60)	(0.65)	(0.27)	(0.74)
Female sex	−0.009	−0.035	n.a.	n.a.	n.a.	n.a.
	(0.07)	(0.04)				
Age (in months)	−0.069***	−0.067***	−0.068***	−0.057***	−0.074***	−0.084***
	(0.01)	(0.01)	(0.01)	(0.01)	(0.01)	(0.01)
Age squared (in months)	0.001***	0.001***	0.001***	0.001***	0.001***	0.001***
	(0.00)	(0.00)	(0.00)	(0.00)	(0.00)	(0.00)
Number of siblings ever born	−0.078***	−0.058***	−0.081***	−0.059*	−0.071***	−0.057***
	(0.01)	(0.01)	(0.02)	(0.03)	(0.02)	(0.02)
Maternal age (log)	0.495***	0.284**	0.612***	0.355	0.423*	0.238
	(0.16)	(0.12)	(0.18)	(0.25)	(0.23)	(0.24)
Maternal primary education	0.076	0.036	0.190***	0.014	0.024	0.066
	(0.05)	(0.06)	(0.06)	(0.07)	(0.07)	(0.09)
Maternal secondary education	0.257***	0.335***	0.307***	0.363***	0.238***	0.320***
	(0.06)	(0.08)	(0.07)	(0.12)	(0.07)	(0.11)
Maternal tertiary education	0.753***	0.620***	0.904***	0.454***	0.602***	0.827***
	(0.06)	(0.14)	(0.17)	(0.13)	(0.12)	(0.18)
Maternal BMI (kg/m^2^)	0.036**	0.044***	0.041**	0.050***	0.033**	0.038***
	(0.01)	(0.01)	(0.02)	(0.01)	(0.02)	(0.01)
Wealth Quintile	0.112***	0.104***	0.088***	0.124***	0.141***	0.089***
	(0.02)	(0.02)	(0.02)	(0.03)	(0.03)	(0.02)
Altitude (log)	−0.298***	−0.190*	−0.384***	−0.188	−0.172	−0.189
	(0.08)	(0.11)	(0.10)	(0.14)	(0.11)	(0.12)
2011 DHS observation	0.038	0.146**	−0.069	0.190*	0.159*	0.094
	(0.06)	(0.07)	(0.07)	(0.10)	(0.08)	(0.09)
Hill	−0.280	0.311*	0.070	−0.186	−0.561***	0.735***
	(.)	(0.16)	(.)	(0.22)	(0.09)	(0.18)
Terai	−0.565***	0.376***	−0.636***	−0.021	−0.438	0.704
	(0.14)	(0.14)	(0.13)	(0.32)	(0.30)	(.)
Urban location	0.092	−0.026	0.181	−0.066	0.036	−0.004
	(0.15)	(0.05)	(0.14)	(0.08)	(0.16)	(0.06)
Constant	−0.762	−1.881**	−1.188	−2.452**	−0.749	−1.198
	(0.74)	(0.81)	(0.89)	(1.19)	(1.19)	(0.93)
Observations (*n*)	3064	3063	1561	1568	1503	1495
*R*-squared	0.180	0.197	0.212	0.209	0.209	0.228

*Note*: Unit of observation = individual child aged 12–59 months. Robust standard errors in parentheses, clustered at the birth year and district levels. All regressions include fixed effects for district (*n* = 75). Omitted month is January and omitted region is Mountains; standard error for Hill and Terai districts is not estimated for some regressions due to multicollinearity. 2011 DHS observation = 1 if the DHS survey was conducted in 2011. Rows 1–7 show NDVI in each trimester of pregnancy and three-month period of the child’s first year, scaled to show difference in HAZ score per 1000-point difference in NDVI. BMI = body mass index; DHS = Nepal Demographic and Health Surveys; n.a. = not applicable; ***p < 0.01, **p < 0.05, *p < 0.1.

**Table 7:** Placebo regression results for predetermined variables affected by NDVI during pregnancy and infancy.

	(1)	(2)	(3)	(4)	(5)	(6)	(7)	(8)
Variable	Age of mother	Maternal primary education	Maternal secondary education	Maternal tertiary education	Maternal BMI (kg/m^2^)	Total children ever born	Wealth (quintile)	Urban location
First trimester	−0.023	−0.202	0.088	0.047	−0.149	0.166	0.074	−0.196**
	(0.04)	(0.13)	(0.10)	(0.04)	(0.52)	(0.32)	(0.34)	(0.09)
Second trimester	−0.081***	0.045	−0.018	0.030	−0.042	−0.219	0.075	−0.180
	(0.03)	(0.07)	(0.08)	(0.02)	(0.49)	(0.40)	(0.24)	(0.13)
Third trimester	−0.011	−0.031	0.142	−0.021	−0.316	−0.070	−0.526*	0.045
	(0.07)	(0.12)	(0.09)	(0.04)	(0.30)	(0.50)	(0.30)	(0.16)
0–2 months	0.019	0.064	0.045	0.018	−0.216	−0.165	−0.211	−0.138
	(0.03)	(0.07)	(0.07)	(0.03)	(0.22)	(0.21)	(0.21)	(0.10)
3–5 months	0.051	0.246**	−0.051	−0.019	0.462	−0.210	−0.388	−0.017
	(0.04)	(0.12)	(0.11)	(0.03)	(0.62)	(0.29)	(0.27)	(0.10)
6–8 months	0.104**	0.048	0.058	0.004	−0.759	0.094	−0.112	−0.053
	(0.04)	(0.06)	(0.06)	(0.03)	(0.54)	(0.45)	(0.25)	(0.16)
9–11 months	0.012	−0.048	−0.025	0.037	0.141	−0.064	0.346	−0.279
	(0.08)	(0.11)	(0.09)	(0.04)	(0.54)	(0.46)	(0.27)	(0.19)
Maternal primary	−0.046***	n.a.	n.a.	n.a.	0.248*	−0.266***	0.336***	0.049*
education	(0.01)				(0.14)	(0.05)	(0.06)	(0.03)
Maternal second-	−0.038***	n.a.	n.a.	n.a.	0.389***	−0.502***	1.052***	0.062**
dary education	(0.01)				(0.12)	(0.05)	(0.05)	(0.03)
Maternal tertiary	0.068***	n.a.	n.a.	n.a.	0.943***	−1.338***	1.382***	0.126**
education	(0.01)				(0.26)	(0.06)	(0.12)	(0.05)
Maternal BMI	0.003***	0.002	0.004**	0.003***	n.a.	−0.015*	0.051***	0.007***
(kg/m^2^)	(0.00)	(0.00)	(0.00)	(0.00)		(0.01)	(0.00)	(0.00)
Total children	0.080***	−0.003	−0.021***	−0.020***	−0.062*	n.a.	−0.082***	−0.002
	(0.00)	(0.00)	(0.00)	(0.00)	(0.03)		(0.01)	(0.00)
Wealth (quintile)	0.007***	−0.013	0.106***	0.024***	0.353***	−0.134***	n.a.	0.093***
	(0.00)	(0.01)	(0.01)	(0.00)	(0.04)	(0.02)		(0.01)
Altitude (log)	0.015**	0.015	0.004	−0.010	0.469***	0.010	−0.099	−0.033
	(0.01)	(0.02)	(0.02)	(0.01)	(0.17)	(0.05)	(0.10)	(0.06)
2011 DHS	0.025*	0.017	0.083***	0.022***	0.649***	−0.192***	−0.193***	−0.050**
	(0.01)	(0.01)	(0.02)	(0.01)	(0.18)	(0.05)	(0.05)	(0.02)
Urban location	0.012**	0.026	0.030	0.020**	0.374***	−0.021	0.712***	n.a.
	(0.00)	(0.02)	(0.02)	(0.01)	(0.13)	(0.06)	(0.09)	
Maternal age	n.a.	−0.275***	−0.191***	0.151***	1.125***	6.401***	0.321***	0.077***
(log)		(0.05)	(0.04)	(0.03)	(0.34)	(0.20)	(0.06)	(0.03)
Constant	2.868***	0.956***	0.616***	−0.514***	14.268***	−17.133***	0.611	0.180
	(0.07)	(0.22)	(0.17)	(0.08)	(1.44)	(0.98)	(0.64)	(0.50)
								
Observations (*n*)	6127	6127	6127	6127	6127	6127	6127	6127
*R*-squared	0.586	0.061	0.252	0.109	0.176	0.617	0.558	0.313

*Note*: Unit of observation = individual child aged 12–59 months. Robust standard errors in parentheses, clustered at the birth year and district levels. All regressions include fixed effects for district (*n* = 75). Omitted month is January. 2011 DHS observation = 1 if the DHS survey was conducted in 2011. Rows 1–7 show NDVI in each trimester of pregnancy and three-month period of the child’s first year, scaled to show difference in HAZ score per 1000-point difference in NDVI. BMI = body mass index; DHS = Nepal Demographic and Health Surveys; n.a. = not applicable; ***p < 0.01, **p < 0.05, *p < 0.1.

## References

[bib0005] Abay K., Hirvonen K. (2016). Does Market Access Mitigate the Impact of Seasonality on Child Growth? Panel Data Evidence from Northern Ethiopia. IFPRI and EDRI Ethiopia Strategy Support Program II Working Paper 85.

[bib0010] Aibar L., Puertas A., Valverde M., Carrillo M.P., Montoya F. (2012). Fetal sex and perinatal outcomes. J. Perinat. Med..

[bib0015] Akresh R., Verwimp P., Bundervoet T. (2011). Civil war, crop failure, and child stunting in Rwanda. Econ. Dev. Cult. Change.

[bib0020] Almond D. (2006). Is the 1918 influenza pandemic over? Long-term effects of *In utero* influenza exposure in the post-1940 U.S. population. J. Polit. Econ..

[bib0025] Andalon M., Azvedo J.P., Rodriguez-Castelan C., Sanfelice V., Valderrama D. (2014). Weather Shocks and Health at Birth in Colombia. World Bank Policy Research Working Paper 7081.

[bib0030] Angrist J.D., Krueger A.B. (2001). Instrumental variables and the search for identification: from supply and demand to natural experiments. J. Econ. Perspect..

[bib0035] Barker D.J. (1995). Fetal origins of coronary heart disease. Br. Med. J..

[bib0040] Black R.E., Victora C.G., Walker S.P., Bhutta Z.A., Christian P., de Onis M., Ezzati M., Grantham-McGregor S., Katz J., Martorell R., Uauy R. (2013). Maternal and child undernutrition and overweight in low-Income and middle-Income countries. Lancet.

[bib0045] Bongaarts J. (2013). The implementation of preferences for male offspring. Popul. Dev. Rev..

[bib0050] Brown M.E., Grace K., Shively G., Johnson K.B., Carroll M. (2014). Using satellite remote sensing and household survey data to assess human health and nutrition response to environmental change. Popul. Environ..

[bib0055] Buckles K.S., Hungerman D.M. (2013). Season of birth and later outcomes: old questions, new answers. Rev. Econ. Stat..

[bib0060] Carlson K. (2015). Fear itself: the effects of distressing economic news on birth outcomes. J. Health Econ..

[bib0065] Checkley W., Gilman R.H., Black R.E., Epstein L.D., Cabrera L., Sterling C.R., Moulton L.H. (2004). Effect of water and sanitation on childhood health in a poor peruvian peri-urban community. Lancet.

[bib0070] Checkley W., Buckley G., Gilman R.H., Assis A.M., Guerrant R.L., Morris K., Valentiner-Branth P., Lanata C.F., Black R.E., The Childhood Malnutrition and Infection Network (2008). Multi-country analysis of the effects of diarrhoea on childhood stunting. Int. J. Epidemiol..

[bib0075] Chodick G., Flash S., Deoitch Y., Shalev V. (2009). Seasonality in birth weight: review of global patterns and potential causes. Hum. Biol..

[bib0080] Coffey D. (2015). Early life mortality and height in indian states. Econ. Hum. Biol..

[bib0085] Coffey, D., M. Geruso, 2015. Sanitation, Disease and Anemia: Evidence from Nepal. Working paper, 13 July 2015.

[bib0090] Cornwell K., Inder B. (2015). Child health and rainfall in early life. J. Dev. Stud..

[bib0095] Cummins J.R. (2015). On the Use and Misuse of Child Height-for-Age Z-score in the Demographic and Health Surveys. Working Paper 201417.

[bib0100] Darrouzet-Nardi A.F. (2015). Child Malnutrition in Farm Households: Three Essays on Agriculture, Health, and Economic Development. PhD Dissertation.

[bib0105] Day F.R., Forouhi N.G., Ong K.K., Perry J.R.B. (2015). Season of birth is associated with birth weight, pubertal timing, adult body size and educational attainment: a UK biobank study. Heliyon.

[bib0110] DiPietro J.A., Voegtline K.M. (2015). The gestational foundation of sex differences in development and vulnerability. Neuroscience.

[bib0115] Fan E., Liu J.-T., Chen Y.-C. (2014). Is the Quarter of Birth Endogenous? Evidence from One Million Siblings in Taiwan. NBER Working Paper 20444.

[bib0120] Hammer J., Spears D. (2016). Village sanitation and child health: effects and external validity in a randomized field experiment in rural India. J. Health Econ..

[bib0125] Headey D.D., Hoddinott J. (2015). Understanding the rapid reduction of undernutrition in Nepal, 2001–2011. PLoS One.

[bib0130] Hoddinott J., Alderman H., Behrman J.R., Haddad L., Horton S. (2013). The economic rationale for investing in stunting reduction. Matern .Child Nutr..

[bib0135] Hu Z., Li T. (2016). Too Hot to Hold: The Effects of High Temperatures During Pregnancy on Birth Weight and Adult Welfare Outcomes. MPRA Paper 68631.

[bib0140] Humphrey J.H. (2009). Child undernutrition, tropical enteropathy, toilets, and handwashing. Lancet.

[bib0145] IFPRI (International Food Policy Research Institute) (2014). Global Nutrition Report 2014: Actions and Accountability to Accelerate the World’s Progress on Nutrition.

[bib0150] IPCC (IIntergovernmenal Panel of Climate Change) (2014). Climate Change 2014: Mitigation of Climate Change. Contribution of Working Group III to the Fifth Assessment Report of the Intergovernmental Panel on Climate Change (No. 5).

[bib0155] Jørkov M.L.S. (2015). Stature in 19th and early 20th century copenhagen: a comparative study based on skeletal remains. Econ. Hum. Biol..

[bib0160] Joshi N.P., Maharjan K.L., Piya L. (2011). Effect of climate variables on yield of major food-Crops in Nepal—A time-Series analysis. J. Contemp. India Stud.: Space Soc..

[bib0165] Krishnamurthy P.K., Hobbs C., Mathiassen A., Hollema S.R., Choularton R.J., Pahari K., Kawabata M. (2013). Climate Risk and Food Security in Nepal—Analysis of Climate Impacts on Food Security and Livelihoods. CCAFS Working Paper 48.

[bib0170] Kumar S., Molitor R., Vollmer S. (2016). Drought and early child health in rural India. Popul. Dev. Rev..

[bib0175] Laidler G.J., Treitz P.M., Atkinson D. (2008). Remote sensing of arctic vegetation: relations between the NDVI, spatial resolution and vegetation cover on boothia peninsula, nunavut. Arct. Inst. N. Am..

[bib0180] Levitas E., Lunenfeld E., Weisz N., Friger M., Har-Vardi I. (2013). Seasonal variations of human sperm cells among 6455 semen samples: a plausible explanation of a seasonal birth pattern. Am. J. Obstet. Gynecol..

[bib0185] Lin A., Arnold B.F., Afreen S., Goto R., Huda T.M.N., Haque R., Raqib R., Unicomb L., Ahmed T., Colford J.M., Luby S.P. (2013). Household environmental conditions are associated with enteropathy and impaired growth in rural Bangladesh. Am. J. Trop. Med. Hyg..

[bib0190] Lokshin M., Radyakin S. (2012). Month of birth and children’s health in India. J. Hum. Resour..

[bib0195] Maccini S., Yang D. (2009). Under the weather: health schooling, and economic consequences of early-life rainfall. Am. Econ. Rev..

[bib0200] Malla G. (2008). Climate change and its impact on nepalese agriculture. J. Agric. Environ..

[bib0205] McKinnish T., Rees D.I., Langlois P.H. (2014). Seasonality in birth defects, agricultural production and urban location. Econ. Hum. Biol..

[bib0210] Mulla Z.D., Plavsic S.K., Ortiz M., Nuwayhid B.S., Ananth C.V. (2013). Fetal sex pairing and adverse perinatal outcomes in twin gestations. Ann. Epidemiol..

[bib0215] NASA (National Aeronautics and Space Administration), 2015. Global Climate Change: Vital Signs of the Planet. http://climate.nasa.gov/evidence.

[bib0220] Nepal CBS (Central Bureau of Statistics) (2004). Nepal Living Standards Survey 2003–2004, NLSS Second Statistical Report, (Vol. 1).

[bib0225] Nepal CBS (Central Bureau of Statistics), 2011 (2011). Nepal Living Standards Survey 2010–2011, NLSS Third Statistical Report, (Vol. 1).

[bib0230] Nepal MoAD (Ministry of Agricultural Development) (2013). Technical Assistance 7762-NEP Preparation of the Agricultural Development Strategy (ADS).

[bib0235] Nepal MoHP (Ministry of Health and Population (2007). Nepal Demographic and Health Survey 2006.

[bib0240] Nepal MoHP (2012). Nepal Demographic and Health Survey 2011.

[bib0245] Nepal NPC (National Planning Commission), 2012. Multi-sector Nutrition Plan for Accelerating the Reduction of Maternal and Child Under-nutrition in Nepal: 2013–2017 (2023). Kathmandu, Nepal.

[bib0250] Osmani S., Sen A. (2003). The hidden penalties of gender inequality: fetal origins of ill-Health. Econ. Hum. Biol..

[bib0255] Panter-Brick C. (1996). Proximate determinants of birth seasonality and conception failure in Nepal. Popul. Stud..

[bib0260] Paxson C., Schady N. (2007). Cognitive development among young children in Ecuador: the roles of wealth, health, and parenting. J. Hum. Resour..

[bib0265] Persson M., Fadl H. (2014). Perinatal outcome in relation to fetal sex in offspring to mothers with pre-gestational and gestational diabetes—a population-Based study. Diabetes Med..

[bib0270] Popkin B.M., Richards M.K., Montiero C.A. (1996). Stunting is associated with overweight in children of four nations that are undergoing the nutrition transition. J. Nutr..

[bib0275] Puentes E., Wang F., Behrman J.R., Cunha F., Hoddinott J., Maluccio J.A., Adair L.S., Borja J.B., Martorell R., Stein A.D. (2016). Early life height and weight production functions with endogenous energy and protein inputs. Econ. Hum. Biol..

[bib0280] Rietveld C.A., Webbink D. (2016). On the genetic bias of the quarter of birth instrument. Econ. Hum. Biol..

[bib0285] Rosenfeld C.S. (2015). Sex-Specific placental responses in fetal development. Endocrinology.

[bib0290] Sassi M. (2015). Seasonality and trends in child malnutrition: time-Series analysis of health clinic data from the dowa district of Malawi. J. Dev. Stud..

[bib0295] Sawaya A.L., Martins P., Hoffman D., Roberts S.B. (2003). The link between childhood undernutrition and risk of chronic diseases in adulthood: a case study of Brazil. Nutr. Rev..

[bib0300] Scholte R., Van den Berg G., Lindeboom M. (2015). Long-Run effects of gestation during the dutch hunger winter famine on labor market and hospitalization outcomes. J. Health Econ..

[bib0305] Schultz-Nielsen M.L., Tekin E., Greve J. (2016). Labor market effects of intrauterine exposure to nutritional deficiency: evidence from administrative data on muslim immigrants in Denmark. Econ. Hum. Biol..

[bib0310] Shively G., Sununtnasuk C., Brown M. (2015). Environmental variability and child growth in Nepal. Health Place.

[bib0315] Sibhatu K.T., Krishna V.V., Qaim M. (2015). Production diversity and dietary diversity in smallholder farm households. Proc. Natl. Acad. Sci. U. S. A..

[bib0320] Skoufias E., Vinha K. (2012). Climate variability and child height in rural Mexico. Econ. Hum. Biol..

[bib0325] Thenkabail P.S., Lyon G.J., Turral H., Biradar C.M. (2009). Remote Sensing of Global Croplands for Food Security.

[bib0330] Tiwari S., Jacoby H.G., Skoufias E. (2013). Monsoon Babies. Rainfall Shocks and Child Nutrition in Nepal. World Bank Policy Research Working Paper 6395.

[bib0335] UNICEF, 2014. The State of the World’s Children 2014 in Numbers: Every Child Counts. http://www.unicef.org/sowc2014/numbers.

[bib0340] UNICEF, 2015. Stop Stunting: South Asia Headline Results—2015 Progress Report. http://www.unicefrosa-progressreport.org/stopstunting.html

[bib0345] United Nations (2013). The Millennium Development Goals Report 2013.

[bib0350] Von Braun J., Kennedy E.T. (1994). Agricultural Commercialization, Economic Development, and Nutrition.

[bib0355] WFP (World Food Programme) (2014). Nepal-Crop Situation Update: A Joint Assessment of the Summer Crops and Outlook of 2013/14 Winter Crops.

[bib0360] WHO (World Health Organization), UNICEF (2009). WHO Child Growth Standards and the Identification of Severe Acute Malnutrition in Infants and Children: A Joint Statement.

[bib0365] Wehby G.L., Castilla E.E., Lopez-Camelo J. (2010). The impact of altitude on infant health in south america. Econ. Hum. Biol..

[bib0370] Weier J., Herring D. (2000). Measuring vegetation (NDVI & EVI). NASA Earth Observatory.

[bib0375] World Bank (2008). Global Monitoring Report 2008: MDGs and the Environment: Agenda for Inclusive and Sustainable Development.

[bib0380] Yamauchi F. (2012). Prenatal seasonality, child growth, and schooling investments: evidence from rural Indonesia. J. Dev. Stud..

