# Supplementary material for: Climatic conditions and child height: Sex-specific vulnerability and the protective effects of sanitation and food markets in Nepal
Source: Econ Hum Biol. 2016 Dec;23:63–75. doi: 10.1016/j.ehb.2016.07.002 (PMC5147727; doi:10.1016/j.ehb.2016.07.002)
Supplement: Supplementary file 1 [file mmc1.docx]

**Supplemental Information (for reviewers only, not for publication)**

**Monthly Histograms of NDVI by Year**

**Figure A.1 Distribution of monthly NDVI in each of the 547 NDHS clusters, February–December 2000**

NDHS = Nepal Demographic and Health Surveys; NDVI = Normalized Difference Vegetation Index.

**Figure A.2 Distribution of monthly NDVI in each of the 547 NDHS clusters, 2001**

NDHS = Nepal Demographic and Health Surveys; NDVI = Normalized Difference Vegetation Index.

**Figure A.3 Distribution of monthly NDVI in each of the 547 NDHS clusters, 2002**

NDHS = Nepal Demographic and Health Surveys; NDVI = Normalized Difference Vegetation Index.

**Figure A.4 Distribution of monthly NDVI in each of the 547 NDHS clusters, 2003**

NDHS = Nepal Demographic and Health Surveys; NDVI = Normalized Difference Vegetation Index.

**Figure A.5 Distribution of monthly NDVI in each of the 547 NDHS clusters, 2004**

NDHS = Nepal Demographic and Health Surveys; NDVI = Normalized Difference Vegetation Index.

**Figure A.6 Distribution of monthly NDVI in each of the 547 NDHS clusters, 2005**

NDHS = Nepal Demographic and Health Surveys; NDVI = Normalized Difference Vegetation Index.

**Figure A.7 Distribution of monthly NDVI in each of the 547 NDHS clusters, 2006**

NDHS = Nepal Demographic and Health Surveys; NDVI = Normalized Difference Vegetation Index.

**Figure A.8 Distribution of monthly NDVI in each of the 547 NDHS clusters, 2007**

NDHS = Nepal Demographic and Health Surveys; NDVI = Normalized Difference Vegetation Index.

**Figure A.9 Distribution of monthly NDVI in each of the 547 NDHS clusters, 2008**

NDHS = Nepal Demographic and Health Surveys; NDVI = Normalized Difference Vegetation Index.

**Figure A.10 Distribution of monthly NDVI in each of the 547 NDHS clusters, 2009**

NDHS = Nepal Demographic and Health Surveys; NDVI = Normalized Difference Vegetation Index.

**Figure A.11 Distribution of monthly NDVI in each of the 547 NDHS clusters, 2010**

NDHS = Nepal Demographic and Health Surveys; NDVI = Normalized Difference Vegetation Index.

**Figure A.12 Distribution of monthly NDVI in each of the 547 NDHS clusters, 2011**

NDHS = Nepal Demographic and Health Surveys; NDVI = Normalized Difference Vegetation Index.

**Figure A.13 Distribution of monthly NDVI in each of the 547 NDHS clusters, January–May 2012**

NDHS = Nepal Demographic and Health Surveys; NDVI = Normalized Difference Vegetation Index.
